# Body image in brace treated and untreated patients: preliminary results from BrAIST

**DOI:** 10.1186/1748-7161-9-S1-O76

**Published:** 2014-12-04

**Authors:** Traci Schwieger, Shelly Campo, Stuart Weinstein, William Clarke, Briana Woods-Jaeger, Kelli Steuber

**Affiliations:** 1 The University of Iowa, Iowa City, Iowa, USA

## Background

There is no consensus regarding the effect of brace treatment on body image in adolescents with adolescent idiopathic scoliosis (AIS).

## Aim

The aim of this study is to compare self-body image, ideal-body image, and self-ideal body image discrepancy, between brace treated and untreated subjects over time.

## Methods

Data from 178 BrAIST subjects that chose treatment were used. Subjects that switched treatment during the study were not included in the analyses. Body image constructs, based on cognitive-behavioral perspectives (1), were used to measure the following: how adolescents’ 1) think they look (self-body image), 2) want to look (ideal-body image), and 3) the difference between these constructs (self-ideal body image discrepancy). The Spinal Appearance Questionnaire (SAQ) (2) measures these constructs and scores were compared with largest curve size, BMI, and quality-of-life measures. Wilcoxon ranked sum tests were conducted to test differences in self-body image means, ideal-body image means, and self-ideal body image discrepancy means between the treated and untreated groups at baseline and at 6, 12, 18, and 24 month follow-up visits.

## Results

At baseline, there were no differences between subjects in the brace group (n=118) and in the observation group (n=60) regarding age, BMI, largest Cobb angle, and quality-of-life. There were no differences at baseline or at any follow-up visit between brace treated and untreated subjects regarding self-body image, ideal-body image, and self-ideal body image. Results from within group analyses found no significant differences within treated or within untreated subjects between baseline body image scores and 24 month body image scores. In addition, there were no significant differences between body image baseline scores and 24 month scores within subjects having < 6◦ degree increase or within subjects having a ≥ 6 ◦ increase in largest Cobb angle.

## Conclusions

This study does not support findings from previous research indicating that wearing a brace has a negative impact on body image. At baseline and follow-up visits, this study found no difference in body image between brace treated and untreated adolescents. In addition, this study found that body image was not impacted after 24 months of brace treatment.

